# Longitudinal comparison of bone, growth, and biochemical markers in breastfed HIV-exposed uninfected and HIV-unexposed Ugandan children

**DOI:** 10.1093/jbmr/zjag024

**Published:** 2026-02-03

**Authors:** Florence Nabwire, Matthew M Hamill, Mary Glenn Fowler, Adeodata Kekitiinwa, Josaphat Byamugisha, Ann Prentice

**Affiliations:** MRC Epidemiology Unit, University of Cambridge, Cambridge CB2 0SL, United Kingdom; MRC Nutrition and Bone Health Research Group, University of Cambridge, Cambridge CB2 0AH, United Kingdom; Formerly based at the MRC Elsie Widdowson Laboratory, Cambridge CB1 9NL, United Kingdom; MRC Nutrition and Bone Health Research Group, University of Cambridge, Cambridge CB2 0AH, United Kingdom; Formerly based at the MRC Elsie Widdowson Laboratory, Cambridge CB1 9NL, United Kingdom; School of Medicine, Johns Hopkins University, Baltimore, MD 21202, United States; School of Medicine, Johns Hopkins University, Baltimore, MD 21202, United States; Baylor College of Medicine Children’s Foundation-Uganda (Baylor-Uganda), Block 5, Mulago Hospital. P.O. Box 72052, Kampala, Uganda; Department of Obstetrics and Gynaecology, Makerere University College of Health Sciences, P.O.Box 7072, Kampala, Uganda; MRC Epidemiology Unit, University of Cambridge, Cambridge CB2 0SL, United Kingdom; MRC Nutrition and Bone Health Research Group, University of Cambridge, Cambridge CB2 0AH, United Kingdom; Formerly based at the MRC Elsie Widdowson Laboratory, Cambridge CB1 9NL, United Kingdom

**Keywords:** HIV-exposed infants, breastfeeding, bone, growth, biochemical markers, tenofovir disoproxil fumarate (TDF)

## Abstract

Due to successful maternal antiretroviral therapy (ART), pediatric HIV infections have declined, leading to a growing population of children HIV-exposed uninfected (CHEU) in Sub-Saharan Africa. However, studies report poorer growth and development in CHEU vs children HIV-unexposed uninfected (CHUU), raising concerns about maternal HIV and ART exposure, particularly tenofovir disoproxil fumarate (TDF). This analysis of data from the Gumba study compares growth trajectories, bone mineral accretion, and biochemical markers of growth and bone metabolism to age 18 mo in Ugandan CHEU, exposed to maternal HIV and TDF-containing ART in utero and via breastmilk (*n* = 85), and CHUU peers (*n* = 80). The groups had similar weight and length 2 wk after birth (WK2), but, despite higher exclusive breastfeeding rates, CHEU had slower growth by WK14 and ~0.5 kg lower body weight and fat mass by WK26. Growth faltering occurred in both groups from WK26, with widening differences: by WK78, 59% of CHEU were stunted compared to 33% of CHUU. Children HIV-exposed uninfected had lower whole-body (WB) BMC and bone area at WK14 and WK26 (*p* = .03 and .0003 for interaction, respectively), but similar WB areal BMD before and after size adjustment, indicating accrued bone mineral was appropriate for their slower growth. There were no group differences in biochemical markers of growth or bone formation. However, CHEU had higher C-terminal telopeptide (CTX), a marker of bone resorption, at WK14 (group difference (95% CI): +50.9 (24.1)%, *p* < .001) coinciding with higher maternal CTX concentrations, and at WK78 (+45.6 (28.9)%, *p* < .05). These findings suggest early onset growth deficits and altered bone metabolism in CHEU by 3 mo of age, possibly linked to the TDF-containing ART initiated during pregnancy and/or disruptions in bone metabolism observed in their mothers. Further studies are needed to investigate the mechanisms and long-term implications for growth and bone health in CHEU.

## Introduction

Globally, 39.9 million people were living with HIV in 2023 of which 53% were women and girls over 15 yr old and 3.5% were children aged 0-14 yr old.^1^ Sub-Saharan Africa (SSA) accounted for 90% of pregnant women and 85% of children aged 0-14 yr living with HIV globally. The current World Health Organization (WHO) guidelines recommend lifelong antiretroviral therapy (ART) for all persons living with HIV, including pregnant and lactating women, for their own health and for prevention of mother-to-child transmission of HIV (PMTCT). WHO also recommends breastfeeding for 12-24 mo for women living with HIV (WLH) while on ART for HIV-free child survival in resource-limited settings.^2^ Globally, 84% of pregnant WLH received ART in 2023.^3^ Since maternal ART for PMTCT guidelines were introduced in 2010, new infections among children have dropped drastically,^1^ and there is now a growing population of HIV-uninfected children exposed to maternal ART in utero and postpartum through breastmilk (children HIV-exposed uninfected, CHEU) in SSA. Despite surviving HIV, CHEU have impaired growth and developmental outcomes compared to peers born to HIV-uninfected mothers (children HIV-unexposed uninfected, CHUU), raising concerns about potential short- and long-term effects of maternal HIV and ART exposure.^4–6^

Tenofovir disoproxil fumarate (TDF), used in the first-line ART regimen for adults in resource-limited settings, including pregnant and lactating WLH, is associated with greater bone loss and fracture risk than ART regimens without TDF.^7–9^ Maternal TDF passes to the fetus via the placenta and to the baby via breastmilk with greater exposure in utero than postpartum via breastmilk.^10–12^ Given the effects in adults,^8^^,^^13^^,^^14^ it is possible that maternal TDF-based ART compromises bone mineral accretion and growth in CHEU. To date, the few studies that have reported bone accretion in CHEU have focused on either in utero or postpartum TDF exposure.^14–16^ Hence, there are limited data on bone outcomes in breastfed CHEU with cumulative in utero and postpartum exposure to maternal TDF-based ART.

We have previously reported in the Gumba [Bone] study of breastfeeding Ugandan WLH initiated on TDF-based ART in pregnancy under the current universal ART guidelines: (1) greater maternal bone loss during lactation and delayed skeletal recovery at 3 mo post-lactation, (2) alterations in maternal bone and calcium-phosphate metabolism, and (3) alterations in breastmilk calcium and phosphorus concentrations.^9^^,^^17^^,^^18^ The current analysis aims to compare growth trajectories, bone mineral accretion, and biochemical makers of growth and bone metabolism in the first 18 mo of life between Ugandan CHEU exposed to maternal HIV and TDF containing ART in-utero and postpartum and CHUU peers born to mothers participating in the Gumba study.

## Materials and methods

### Study details

Participants were infants born to mothers enrolled in the 3-yr*,* prospective observational cohort study (Gumba study), which recruited 100 WLH and 100 women without HIV (REF) during pregnancy at the former midwife-led antenatal clinic at Mulago National Referral Hospital in Kampala, Uganda between January 2015 and September 2017. All WLH had been newly diagnosed with HIV and initiated onto first-line ART regimen consisting of TDF-lamivudine (3TC) and efavirenz (EFV) during the index pregnancy, in line with the WHO and national guidelines at the time.^19–21^ All REF women were ART-naïve and remained HIV-negative during the study. Detailed description of the Gumba study, maternal characteristics and outcomes (anthropometry, bone measures, body composition, breast milk composition, and biochemical markers) have been published elsewhere.^9^^,^^17^^,^^18^

Informed consent in the Gumba study was obtained antepartum for mother-infant dyads before study enrolment, in line with prevailing the Uganda National Council for Science and Technology (UNCST) guidelines for research involving children. All pregnant women gave written consent in Luganda or English for themselves and the index infant by either signing or appending their left thumb on the consent form, after attending 2 informed consent sessions with a trained study counselor. Informed consent was also obtained from the husband/child’s father, if available, before study enrolment. Exclusions for infants included: preterm birth <37 wk gestation and neonatal death before 2 wk. Gumba study protocol was reviewed and approved by The Joint Clinical Research Centre Ethics Review Committee, Mulago Hospital Insitutional and Ethics Review Board, and UNCST.

Study measurements on the infants were conducted at 2, 14, 26, 52, and 78 wk of age (WK2, WK14, WK26, WK52, and WK78, respectively). The prevailing national PMTCT and early infant HIV diagnosis (EID) guidelines released in early 2013 based on WHO guidance, recommended WLH to breastfeed their infants for at least 12 mo while on ART.^19^^,^^20^ The guidelines also recommended HIV testing and clinical monitoring of CHEU until 18 mo of age with the first HIV DNA polymerase chain reaction (PCR) test at 6 wk of age, a repeat PCR test at 6 wk after cessation of breastfeeding, and a final confirmatory HIV antibody rapid test at 18 mo.^19^^,^^21^ Infants who tested HIV positive at any point in the EID cascade were immediately recommended to start on pediatric ART. All CHEU infants in the study were provided with nevirapine syrup (antiretroviral drug) for HIV prophylaxis from birth until the first PCR test at 6 wk of age, and cotrimoxazole prophylaxis from 6 wk of age until the results of the second PCR test were known.^19^^,^^21^

### Anthropometry, growth, and infant characteristics

Weight, recumbent length, mid-upper arm circumference, and head circumference were obtained at every visit following standard procedures.^22^ Infants were weighed without clothes (or in light clothing during the rainy/cold season) using a digital infant weighing scale (SECA 374, manufacturer SECA GmbH). The weighing scale was calibrated daily following a standard protocol. Recumbent length was measured using a standard wooden length board supplied by UNICEF. Standard tapes (SECA 212; manufacturer SECA GmbH) were used to measure head circumference following standard procedures. Mid-upper circumference (MUAC) was measured on the left arm as per standard procedures using UNICEF color coded tapes for children aged 6-59 mo. Z-scores for growth indices (weight-for-age (WAZ), length-for-age (LAZ), weight-for-length (WFLZ), mid-upper arm circumference-for-age (MUACZ), and head circumference-for-age (HCAZ)) were generated based on WHO 2010 growth standards in WHO Anthro software plugin for STATA.^23^ Children with WAZ, LAZ, and WFLZ less than −2 were categorized as underweight, stunted, and wasted, respectively.^22^ Data on mode of birth and birth weight were obtained from the maternity discharge forms, where possible.

### Bone measures using DXA

At WK2, WK14, and WK26, the DXA scanner (Model: Discovery W by Hologic, Inc.) at the Makerere University Johns Hopkins University (MUJHU) Research Center at Mulago Hospital was used to measure infant whole body and LS BMC (g), bone area (BA, cm^2^), areal BMD (aBMD, g/cm^2^), and total mass (g), lean mass (g), fat (g), and percentage fat (%) from whole body scan. The automated infant whole-body (WB) and LS scanning modes (Apex software version 12.3.3; Hologic Inc.) were used to acquire scans with the infant in a supine position without sedation, while sleeping. Infants who woke partway through scanning were taken off the DXA, breastfed and soothed to sleep before a second attempt. If the second attempt failed, the infant was rescheduled for a repeat scan within 1 wk. At each study timepoint, a maximum of 3 tries was attempted to acquire scans at each site. The long-term stability of the DXA aBMD measurements during the study, determined using daily phantom calibration procedures, was ±0.5%. This was within the allowed range of ±1.5% for Hologic densitometers.^24^^,^^25^

DXA images were scrutinized and analyzed using Hologic Apex software (version 5.6.0.4), and poor-quality scans were excluded. All infant WB scans were analyzed with head to avoid longitudinal inconsistencies in placement of the head region box and overlap of the chin and neck due to small body size and short neck region at WK2. For LS scans, the L1-L4 region was analyzed. Local SD-scores of DXA variables were calculated for CHEU using the means and standard deviations for CHUU at each timepoint.

### Biological samples: collection, processing, and laboratory analysis

At WK14, WK26, WK52, and WK78, non-fasted venous blood (2 mL) was collected from the arm into plain serum tubes, following standard laboratory procedures for blood collection in children at the Baylor-Uganda Clinical Center of Excellence for Pediatric HIV. All blood samples were centrifuged, aliquoted and stored at −80 °C in Uganda and later airfreighted on dry ice, in batches, for storage and laboratory analysis at the MRC Elsie Widdowson Laboratory and the MRC Epidemiology Unit in Cambridge, UK.

Serum samples were analyzed for vitamin D status (25OHD), hormones involved in growth and bone metabolism: intact insulin-like growth factor 1 (IGF1), IGF-binding protein 3 (IGFBP3) and bone turnover markers: procollagen type 1 N-terminal-propeptide (P1NP), C-terminal telopeptide (CTX), and osteocalcin (OC). The index IGF1/IGFBP3 is used as a marker of growth hormone deficits and is presented as a molar ratio obtained from data in μg/L by multiplying IGF1 by 0.13 and IGFBP3 by 0.035.^26^^,^^27^ P1NP is regarded as a bone formation marker, CTX as a bone resorption marker, and circulating OC as a mix of osteoblastic factors released during formation and related fragments released during resorption. The ratio of P1NP/CTX is used as an index of the balance between bone formation and bone resorption.^28^

Full details of the collection processes and assays are given in Supplement S1.

### Questionnaires: infant health status, lifestyle, and feeding practices

Standardized questionnaires administered to the mother in Luganda and English, were used at each visit to collect data on maternal socio-economic characteristics and lifestyle, infant medical history and health status, and feeding practices at all visits. The medical history questionnaire collected information on whether the infant had been ill since the last visit, type of illness and duration, and any treatment/medication received based on maternal report. A locally-adapted WHO semi-structured questionnaire^29^ was used to collect information on breastfeeding status and frequency, and whether any foods, drinks, and medication were offered to the infant since the last visit. At WK52 and WK78, more detailed information on the infant diet was collected about the number of meals and milk feeds, and a listing of all foods and drinks consumed the previous day. Breastmilk and family foods/drinks were assigned into 8 groups: breastmilk (yes/no), grains, roots, tubers and plantains (yes/no), legumes and nuts (yes/no), milk and dairy products (yes/no), flesh foods (yes/no), eggs (yes/no), vitamin A rich fruits and vegetables (yes/no), and other fruits and vegetables (yes/no).^29^

WHO guidance and definitions were used to characterize feeding practices. Exclusive breastfeeding was defined as breastfeeding with no other food or drink, including water, but including vitamins and prescribed medication.^29^ For feeding practices at WK52 and WK78, infants who consumed foods and beverages from at least 5 out of the 8 food groups were classified as having met the recommended minimum dietary diversity (MDD); and those who consumed solid, semi-solid, or soft foods (including animal milk feeds for non-breastfed infants) the recommended number of times for their age or more, were classified as having met the minimum meal frequency (MMF). For breastfed infants, the recommended MMF is 1-2 complementary foods/meals per day for 6- to 8-mo-olds, and 3-4 complementary foods/meals plus 1-2 nutritious snacks per day for 9- to 23-mo-olds. Minimum meal frequency recommendation for non-breastfed children aged 6-23 mo is 4-5 meals per day including at least 2 animal milk feeds (fresh or fermented milk or yoghurt). Infants who achieved both MDD and MMF were classified as having received the recommended minimum acceptable diet (MAD).^29^

### Statistical methods

Statistical analysis used DataDesk 8.3 software (Data Description Inc.). Descriptive statistics are expressed as means (SD) for normally distributed data, geometric means (25th percentile, 75th percentile) calculated by back-transformation of log_e_ values for positively-skewed data, medians (25th percentile, 75th percentile) for categorical data, and percentages (%) for proportions. Thresholds of serum 25OHD of 25 nmol/L^30^ and 50 nmol/L^31^ were used to provide prevalence estimates of low vitamin D status.

General Linear Models were developed in DataDesk to test for differences between CHEU and CHUU at each timepoint, and changes between timepoints within each group, which use hierarchical/nested repeated-measures ANOVA and ANCOVA as appropriate. The model for each variable included an individual identifier (ID, nested by group), timepoint, group, and a group^*^timepoint interaction term. Scheffé post-hoc tests were used to account for multiple testing and to provide estimates (expressed as means (95% CI)) of the size and significance of group differences at each time point and within-group changes between timepoints. Potential covariates were included as discrete or continuous variables as necessary. Where the interaction term was not significant, it was removed to give an estimate of the overall difference between groups. However, this made little or no difference to the significance of the group and timepoint effects in the linear models and are not presented. In addition, adjusting for age in the statistical models rather than timepoint did not affect the differences materially between groups and are also not presented.

The models for anthropometric, 25OHD, and DXA variables were for 5 (WK2-WK78), 4 (WK14-WK78), and 3 (WK2-WK26) timepoints respectively, using absolute values of the measures. The models for the biochemical markers of growth and bone turnover were for 4 timepoints (WK14-WK78), with data transformed into natural logarithms (log_e_) to normalize positively-skewed distributions and to compare relative size effects between the biochemical markers as sympercents ([difference/mean] × 100%).^32^ To consider the effects of differences in bone and body size on bone mineral accretion, aBMD was analyzed before and after size adjustment by adding BA, weight, and length to the model.^33^ As the General Linear Model software in DataDesk does not impute missing values, each longitudinal model was restricted to infants who provided at least 2 useable measures at different timepoints across the study. To consider the potential impact of missing values, sensitivity models were run for all variables by restricting the analyses to those infants with values at all relevant timepoints.

Infant age, sex, exclusive breastfeeding (yes/no), maternal prepartum parity (nulliparity/parity ≥1), and prior use of depot medroxyprogesterone acetate (DMPA, yes/no) were investigated as potential explanatory variables by adding each to the models as an independent factor nested within the ID along with group. As these analyses were exploratory and not primary outcomes, the effect sizes and significances are not described.

The 25OHD concentration of Gumba mothers was shown to be higher during Kampala’s wet seasons (Mar-May, Sept-Dec) than during dry seasons (January-February, June-August).^17^ A similar seasonal variation was observed in the infants (wet-dry: mean (95% CI) = +5.7 (3.4) nmol/L, *p* = .0009, 4-timepoint model). The size and significance of group differences were not materially affected by adjustment for season, and are not discussed further.

A two-tailed *p*-value of ≤.05 was considered significant for all tests.

## Results

The flow of infants through the study is illustrated in Supplement S2, which gives the reasons for loss to the study. One infant had a positive DNA PCR at 6 wk and confirmed HIV positive by WK14. Data from this infant were excluded from analysis. No other CHEU had positive tests. The total numbers of infants delivered at term that participated in the study on at least 2 occasions and therefore included in the longitudinal data analyses were CHEU = 85 and CHUU = 80. The numbers of useable datapoints collected at each visit from these participants are itemized by measurement category in Supplement S3. The number and distribution of missing data points were broadly similar between CHEU and CHUU and was due to non-attendance at scheduled visits or poor-quality measures for anthropometry and DXA, and to insufficient/no sample for biochemistry.

Table 1 presents the maternal, prenatal, and birth characteristics for the infants. Table 2 gives their age and anthropometric variables at each measurement timepoint. Age at each timepoint was similar between the groups except CHEU were younger on average at WK78. Table 2 also lists the proportions of infants who were below or above the nutritional status thresholds. There were few differences in either the maternal or infant characteristics at birth or at WK2.

**Table 1:** Maternal, prenatal and birth characteristics of Gumba infants.

**Variable**	**CHEU** **(*n* = 85)**	**CHUU** **(*n* = 80)**	***p***
**Maternal characteristics at enrolment**			
**Age**^**a**^**, years**	24.5 (4.3)	24.4 (4.7)	.73
**Parity**^**b**^	1 (0, 2)	0 (0, 1)	.008
**Nulliparity**^**c**^**, %**	35.3	53.8	.017
**Prior DMPA**^**c**^**, %**	40.2	19.2	.003
**Maternal characteristics at 36 wk pregnancy**			
**Time since ART initiation, weeks**	10.8 (5.0)	-	
**ART adherence, %**	99.1 (1.8)	-	
**CD4 count, cells/mm^3^**	424 (195)	-	
**Weight**^**a**^**, kg**	65.6 (9.4)	68.6 (11.7)	.07
**Height**^**a**^**, m**	1.57 (0.05)	1.58 (0.06)	.17
**Infant birth characteristics**			
**Sex male**^**c**^**, %**	47.1	51.3	.59
**Birthweight**^**a,d**^**, kg**	3.28 (0.50)	3.34 (0.55)	.50
**Gestational age**^**a**^**, weeks**	40.9 (2.0)	40.8 (1.7)	.75

Data are means (SD) for continuous variables, medians (25th and 75th percentiles) for categorical variables, and percentages for proportions. Data are for infants who attended ≥2 measurement sessions in the study. Statistical comparisons were by ^a^2-tailed *t*-test, ^b^Wilcoxin rank sum test, or ^c^2-sample z-test for proportions. ^d^Birth weight obtained from maternity discharge forms where available: CHEU *n* = 75 and CHUU *n* = 68.

Abbreviations: ART, antiretroviral therapy; DMPA, depot medroxyprogesterone acetate; CHEU, children HIV-exposed uninfected; CHUU, children HIV-unexposed uninfected.

**Table 2:** Age, anthropometry, and breast-feeding of Gumba infants.

	**WK2**		**WK14**		**WK26**		**WK52**		**WK78**	
	**CHEU** **(*n* = 81)**	**CHUU** **(*n* = 75)**	**CHEU** **(*n* = 83)**	**CHUU** **(*n* = 79)**	**CHEU** **(*n* = 75)**	**CHUU** **(*n* = 73)**	**CHEU** **(*n* = 68)**	**CHUU** **(*n* = 64)**	**CHEU** **(*n* = 56)**	**CHUU** **(*n* = 66)**
**Age, weeks**	2.2 (0.5)	2.1 (0.4)	14.3 (0.5)	14.2 (0.8)	26.5 (0.8)	26.5 (0.7)	52.5 (1.3)	52.6 (0.8)	76.8 (3.5)^a^	78.4 (1.8)
**Weight, kg**	3.61 (0.54)	3.65 (0.50)	6.10 (0.80)^b^	6.49 (0.83)	7.34 (1.08)^a^	7.78 (1.02)	8.81 (1.09)^b^	9.22 (1.22)	9.75 (0.96)^a^	10.37 (1.16)
**Length, cm**	51.3 (2.4)	51.6 (2.2)	60.2 (2.6)^c^	61.4 (2.9)	65.6 (2.5)	66.4 (2.6)	70.9 (3.4)^b^	72.5 (3.2)	75.4 (3.3)^a^	77.6 (3.1)
**MUAC, cm**	-	-	-	-	14.0 (1.2)^c^	14.4 (1.2)	14.7 (1.1)	14.8 (1.1)	15.0 (1.1)	15.1 (1.0)
**HC, cm**	36.4 (1.6)	36.4 (1.3)	40.8 (1.4)	41.1 (1.7)	43.1 (1.8)	43.3 (1.5)	45.7 (1.5)	46.1 (1.4)	46.9 (1.4)^c^	47.6 (1.4)
**WAZ**	−0.26 (0.98)	−0.12 (1.02)	−0.29 (1.01)^a^	0.20 (1.04)	−0.41 (1.18)^a^	0.06 (1.11)	−0.53 (1.08)	−0.18 (1.15)	−0.65 (0.84)^c^	−0.27 (0.96)
**LAZ**	−0.43 (1.19)	−0.22 (1.15)	−0.50 (1.10)^a^	0.09 (1.16)	−0.54 (1.01)	−0.21 (1.10)	−1.62 (1.33)	−1.02 (1.32)	−2.01 (1.23)	−1.45 (1.22)
**WFLZ**	−0.17 (1.37)	−0.23 (1.18)	0.19 (1.19)	0.34 (1.30)	−0.03 (1.34)	0.36 (1.15)	0.39 (1.31)	0.42 (1.46)	0.39 (1.03)	0.56 (1.18)
**MAZ**	-	-	-	-	−0.06 (1.08)^c^	0.26 (0.99)	0.21 (0.96)	0.31 (0.94)	0.30 (0.95)	0.35 (0.83)
**HCZ**	0.64 (1.30)	0.71 (1.06)	0.39 (1.08)	0.63 (1.29)	0.23 (1.44)	0.22 (1.50)	0.15 (1.11)	0.40 (1.03)	0.18 (1.02)	0.49 (0.93)
**Underweight, %**	6.2	4.0	8.4	2.5	10.7	4.1	10.4	7.8	7.4	4.7
**Stunted, %**	8.6	6.7	7.2	3.8	6.7	5.5	40.9	26.6	59.3^d^	32.8
**Wasted %**	11.1	6.7	4.8	3.8	8.0	1.4	6.0	4.7	0	0
**Overweight, %**	0	2.7	0	3.8	1.3	2.7	1.5	1.6	0	1.6
**Breastfed, %**	100	100	97.6	100	93.3	97.3	58.8^d^	87.5	0^d^	43.9
**EBF, %**	84.0^d^	58.7	88.0^d^	67.1	80.0^d^	45.2	-	-	-	-
**Had been ill, %**	8.6	21.3^e^	27.7	43.6 ^d^	37.8	46.5	40.3	67.2^d^	25.0	53.0^d^

Data are for infants who were measured on ≥2 occasions (*n* = 165, CHEU 85, CHUU 80; infants measured at 5 visits = 61%, (CHEU 57%, CHUU 66%), 4 visits = 21% (CHEU 22%, CHUU 19%), 3 visits = 12% (CHEU 14%, CHUU 10%), and 2 visits = 6% (CHEU 7%, CHUU 5%). Continuous variables are summarized as means (SD), categorical variables as percentages.

a-c Significance of difference between groups at each timepoint ^a^*p* ≤ .001, ^b^*p* ≤ .01, and ^c^*p* ≤ .05 from pairwise Scheffé post-hoc tests obtained in hierarchical 5-timepoint models with timepoint, group infant ID nested in group and a timepoint^*^group interaction term. Differences between groups at each timepoint from the same models are summarized in Table 4 and Supplement S6, and within-individual changes between timepoints in Supplement S7.

d and e Significance of difference by 2-tailed z-test for proportions ^d^*p* ≤ .005 and ^e^*p* = .03.

Variables with missing values are age: CHEU at WK52 *n* = 1, CHEU at WK78 *n* = 2, and CHUU at WK78 *n* = 2; HC: CHEU at WK2 *n* = 1, CHUU at WK2 *n* = 3, CHUU at WK26 *n* = 1, and CHUU at WK78 *n* = 1; HCAZ: CHEU at WK2 *n* = 1, CHUU at WK2 *n* = 3, CHEU at WK26 *n* = 1, CHUU at WK26 *n* = 1, CHEU at WK52 *n* = 1, CHEU at WK78 *n* = 2, and CHUU at WK78 *n* = 3; Length: CHEU at WK52 *n* = 1; MUAC: CHUU at WK26 *n* = 2; MAZ: CHUU at WK26 *n* = 2, CHEU at WK52 *n* = 1, CHEU at WK78 *n* = 2, and CHUU at WK78 *n* = 2; WAZ: CHEU at WK52 *n* = 1, CHEU at WK78 *n* = 2, and CHUU at WK78 *n* = 2; LAZ: CHEU at WK52 *n* = 2, CHEU at WK78 *n* = 2, and CHUU at WK78 *n* = 2; WFLZ: CHEU at WK52 *n* = 1. MUAC was not measured at WK2 or WK14.

Abbreviations: CHEU, children HIV-exposed uninfected; CHUU, children HIV-unexposed uninfected; EBF, exclusively breastfed, HC, head circumference; HCAZ, head circumference-for-age SD score; LAZ, length-for-age SD-score; MAZ, MUAC-for-age SD score; MUAC, mid-upper arm circumference; WAZ, weight-for-age SD-score; WFLZ, weight-for-length SD-score; WK2, WK14, WK26, WK52, and WK78, 2, 14, 26, 52, and 78 wk of age, respectively.

The majority of infants were breast-fed in the first 6 mo but more CHEU were exclusively breastfed in the first 6 mo (Table 2). By WK52, a greater proportion of CHEU had stopped breastfeeding, and none were breastfed at WK78 in contrast to 44% of CHUU who continued to receive some breastmilk. There was no evidence of a sex difference in the proportion of infants who were exclusively breastfed. The dietary intakes and diet quality of the infants at WK52 and WK78 are described in Supplement S4. At WK26, only 37% of infants (CHEU = 20%, CHUU = 55%) were given some foods and drinks. Overall, diet quality was poor in both groups. However, more CHEU were not receiving the recommended MMF or MAD at WK78, because they had stopped breastfeeding but did not receive animal milk feeds as per WHO recommendations for non-breastfed children. Regarding health status, CHEU had fewer episodes of illness compared to CHUU in the first 18 mo of life based on maternal reports (Table 2). Respiratory tract infections (mostly coughs, colds, and flu) were the most frequently reported illnesses, accounting for 62.1% of all episodes of illness, followed by malaria (11.3%) and diarrhea (7.0%).

**Figure 1:**
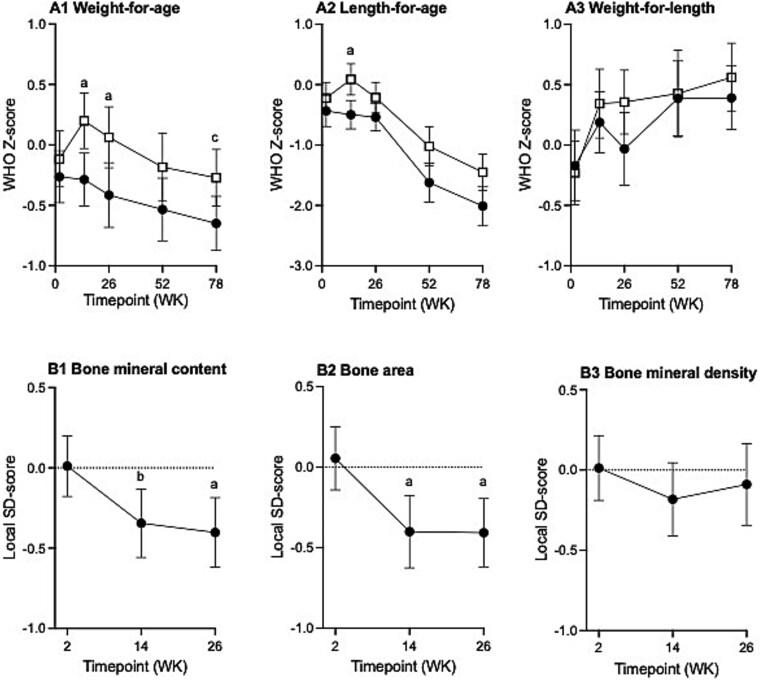
Growth and whole-body (WB) bone indices of Gumba infants. Data are means ±95% CIs. Symbols: black rounds, children HIV-exposed uninfected (CHEU); white squares, children HIV-unexposed uninfected (CHUU). The numbers of children measured at each timepoint can be found in Tables 2 and 3. Panel (A1-3) Growth indices for both groups expressed as SD-scores (Z) against the WHO reference. Significance of difference between groups from pairwise Scheffé tests obtained from hierarchical repeated measures ANOVA models with timepoint, group, infant ID nested within group and timepoint^*^group interaction (Table 4, Supplement S4): a, *p* ≤ .001; b, *p* ≤ .01; c, *p* ≤ .05. Significance of within-individual changes by group are in Supplement S7. Panel (B1-3) DXA WB bone variables for CHEU expressed as SD-scores (local) against CHUU values at each timepoint. Significance of difference from zero at each timepoint obtained from one-sample *t*-tests (Supplement S5): a, *p* ≤ .001; b, *p* ≤ .01; c, *p* ≤ .05.

The patterns of somatic and bone growth differed between the two groups. In addition to Table 2, the anthropometric and DXA data by timepoint are presented in Tables 3 and 4 and Supplement S5. The group at each timepoint from the longitudinal models are presented in Table 4 and Supplement S6, and within-individual changes between timepoints in Supplement S7. Figure 1A illustrates the changes in WAZ, LAZ, and WFLZ over time by group. Figure 1B illustrates the changes over time by group in WB BMC, BA, and aBMD expressed as local SD-scores using CHUU as reference.

**Table 3:** DXA bone measures and body composition of Gumba infants in the first 6 mo.

	**WK2**		**WK14**		**WK26**	
	**CHEU**	**CHUU**	**CHEU**	**CHUU**	**CHEU**	**CHUU**
**WB bone measures**	*n* = 69	*n* = 69	*n* = 76	*n* = 75	*n* = 69	*n* = 69
**BMC, g**	79.5 (14.0)	79.2 (17.7)	132.3 (19.1)^a^	139.2 (20.1)	167.0 (22.4)^a^	174.6 (24.4)
**BA, cm^2^**	420.7 (42.7)	417.6 (52.3)	611.3 (55.9)^a^	633.6 (55.8)	704.0 (58.7)^a^	730.7 (64.7)
**aBMD, g/cm^2^**	0.188 (0.019)	0.188 (0.023)	0.216 (0.017)	0.219 (0.016)	0.237 (0.018)	0.238 (0.017)
**LS bone measures**	*n* = 77	*n* = 74	*n* = 80	*n* = 79	*n* = 65	*n* = 69
**BMC, g**	1.74 (0.30)^b^	1.88 (0.30)	2.27 (0.39)	2.34 (0.38)	2.79 (0.45)	2.85 (0.42)
**BA, cm^2^**	8.73 (0.90)^b^	9.15 (0.82)	11.16 (1.06)^a^	11.68 (0.89)	12.59 (1.34)	12.81 (1.10)
**aBMD, g/cm^2^**	0.199 (0.029)	0.205 (0.025)	0.203 (0.024)	0.199 (0.025)	0.221 (0.021)	0.222 (0.022)
**Body composition**	*n* = 69	*n* = 67	*n* = 76	*n* = 75	*n* = 69	*n* = 69
**Lean mass, g**	2929 (400)	2946 (472)	3563 (625)^c^	3788 (836)	4515 (1010)	4499 (1091)
**Fat mass, g**	1081 (343)	1101 (413)	2972 (884)	3130 (960)	3285 (1190)^a^	3773 (1296)
**% Fat**	26.7 (7.1)	26.8 (8.5)	44.9 (10.3)	44.8 (11.6)	41.6 (13.2)	45.2 (12.9)

Data are expressed as means (SD) and are for infants who were scanned successfully on ≥2 occasions for WB *n* = 152 (CHEU 77, CHUU 75); infants with 3 scans = 74%, (CHEU 70%, CHUU 79%) and 2 scans = 26%, (CHEU 30%, 21%) and for LS *n* = 161, (CHEU 82, CHUU 79); infants with 3 scans = 76% (CHEU 73%, CHUU 27%) and 2 scans = 24% (CHEU 27%, CHUU 20%). Body composition variables were from WB scans.

There are no variables in each category with missing data.

Significance of difference between groups at each timepoint: ^a^*p* ≤ .001, ^b^*p* ≤ .01, ^c^*p* = .06 from pairwise Scheffé post-hoc tests obtained in hierarchical 3-timepoint repeated measures models with timepoint, group, infant ID nested in group and a timepoint^*^group interaction. Differences between groups at each timepoint from the same models are given in Table 4 and within-individual changes between timepoints in Supplement S7.

Abbreviations: aBMD, areal BMD; BA, bone area; CHEU, children HIV-exposed uninfected; CHUU, children HIV-unexposed uninfected; LS, lumbar spine L1-4; WB, whole-body with head; WK2, WK14, and WK26, 2, 14, and 26 wk of age, respectively.

**Table 4:** Differences between CHEU and CHUU at each timepoint: anthropometry and DXA measures.

	**WK2**	**WK14**	**WK26**	**WK52**	**WK78**	***p* for interaction**	***p* for group**	***p* for timepoint**
**Anthropometry**								
**Weight, kg**	−0.06 (0.18)	−0.35 (0.18)^b^	−0.42 (0.19)^a^	−0.38 (0.20)^b^	−0.56 (0.21)^a^	.01	.007	<.0001
**Length, cm**	−0.27 (0.63)	−1.03 (0.62)^c^	−0.90 (0.66)	−1.35 (0.71)^b^	−1.67 (0.74)^a^	.06	.004	<.0001
**MUAC, cm**	-	-	−0.31 (0.22)^c^	−0.25 (0.24)	−0.15 (0.25)	.64	.17	<.0001
**HC, cm**	−0.06 (0.32)	−0.29 (0.31)	−0.25 (0.33)	−0.39 (0.35)	−0.60 (0.37)^c^	.30	.12	<.0001
**WB bone measures**								
**BMC, g**	−1.7 (3.8)	−7.8 (3.6)^a^	−8.0 (3.8)^a^	-	-	.03	.05	<.0001
**BA, cm^2^**	+0.5 (10.6)	−24.4 (10.0)^a^	−27.9 (10.6)^a^	-	-	.0003	.04	<.0001
**aBMD, g/cm^2^**	−0.002 (0.004)	−0.004 (0.004)	−0.002 (0.004)	-	-	.70	.34	<.0001
**LS bone measures**								
**BMC, g**	−0.13 (0.08)^b^	−0.06 (0.08)	−0.07 (0.09)	-	-	.43	.08	<.0001
**BA, cm^2^**	−0.38 (0.23)^b^	−0.53 (0.22)^a^	−0.26 (0.25)	-	-	.30	.005	<.0001
**aBMD, g/cm^2^**	−0.006 (0.006)	+0.003 (0.006)	−0.001 (0.006)	-	-	.06	.70	<.0001
**Body composition**								
**Lean mass, g**	−34 (209)	−237 (194)^d^	0 (207)	-	-	.20	.38	<.0001
**Fat mass, g**	−18 (246)	−174 (228)	−484 (243)^a^	-	-	.03	.07	<.0001
**% Fat**	+0.3 (3.1)	+0.1 (2.9)	−3.7 (3.1)	-	-	.13	.41	<.0001

Data are mean differences (95% CI) from pairwise Scheffé post-hoc tests obtained in hierarchical models with timepoint, group, infant ID nested in group, and a timepoint^*^group interaction term. For models, where the interaction term was not significant, removing the interaction term made no material effect to the *p*-values for group or timepoint. Data are for infants who were measured on ≥2 occasions in each category, the values and numbers are given in Tables 2 and 3. Differences between groups in anthropometric SD-scores are in Supplement S5.

a-d Significance of difference between groups at each timepoint ^a^*p* ≤ .001, ^b^*p* ≤ .01, ^c^*p* ≤ .05, ^d^*p* = .06. Within-individual changes between timepoints from the same models are summarized in Supplement S7.

Abbreviations: aBMD, areal BMD; BA, bone area; CHEU, children HIV-exposed uninfected; CHUU, children HIV-unexposed uninfected; HC, head circumference; LS, lumbar spine L1-4; MUAC, mid-upper arm circumference; WB, whole-body with head; WK2, WK14, WK26, WK52, and WK78, 2, 14, 26, 52, and 78 wk of age, respectively.

The mean weights and lengths of CHUU were close to the international standards in the first 6 mo (Table 2, Figure 1A). Those of CHEU were similar to CHUU at WK2, but CHEU infants were significantly lighter and shorter at WK14 and subsequent timepoints (*p*: weight = 0.007, length = 0.004 for group (Table 4)). Both groups exhibited growth faltering relative to the international standards from WK26 (Table 2, Figure 1A), but the differences between CHEU and CHUU in weight and length tended to increase across time (*p*: weight = 0.01, length = 0.06 for interaction (Table 4)). The group difference was most marked in length at WK78 (an average in the longitudinal model of −1.7 cm, *p* = .0008, Table 4), with 59% of CHEU classified as stunted compared to 33% of CHUU (*p* = .003, Table 2). Children HIV-exposed uninfected had lower MUAC and MAZ at WK26 (*p* = .02 (Table 2) and *p* = .03 (Supplement S5), respectively) but not at later timepoints. Mean weight-for-length in both groups was at or higher than the international standard at most timepoints after WK2 (Table 2, Figure 1A), and only a few infants in either group were classified as wasted at any timepoint (Table 2).

Whole-body BMC and BA were similar in the 2 groups at WK2 but CHEU had lower values at WK14 and WK26, indicating slower bone growth (*p*: BMC = 0.03, BA = 0.0003 for interaction Table 4 and Figure 1B). There were no significant group differences in WB aBMD before or after size-adjustment, indicating the group differences in WB bone mineral accretion were appropriate for their differences in growth. Unlike the WB, CHEU had lower LS BMC and BA values at WK2 but these differences diminished by WK26 and there was no evidence of a group difference in growth pattern for these 2 variables (*p* = .43 and .30 for interaction, respectively, Table 4). However, there was a trend toward a group difference in the within-individual pattern of change in LS aBMD between WK2 and WK26 (*p* = .06 for interaction, Table 4), also observed after size-adjustment (*p* = .04 for interaction, data not shown).

At WK2 lean and fat masses from the WB DXA scans were similar between the two groups (Table 2). Lean mass tended to be lower in CHEU than CHUU at WK14 but the within-individual pattern of change from WK2 to WK26 was not significantly different between the groups (*p* = .20 for interaction, (Table 4)). In contrast, CHEU had slower increases in fat mass than CHUU during that time (*p* = .03 for interaction, Table 4, Supplement S7), such that CHEU infants had less fat mass at WK26 by an average of −0.48 kg from the longitudinal model (*p* < .0001, Table 4).

Females had lower weight and length than males at WK2, the differences increasing during infancy but diminishing by WK78. Similar sex differences were observed for BMC, BA, and lean mass to WK26. Infants that were exclusively breastfed had greater weight, BMC, BA, and fat mass at the same timepoint than infants who were mixed-fed. There was no evidence of an effect of infant sex, exclusive breastfeeding, maternal parity, or maternal use of DMPA (prior and postpartum) on differences between CHEU and CHUU, or in their trajectories of growth and bone accretion over time. Adjustment for these variables made little or no difference to the coefficients in the models.

The biochemical data and results from the longitudinal models are presented in Tables 5 and 6 and Supplement S8 at the 4 timepoints when bloods were collected. The vitamin D status of CHEU was good, with mean 25OHD concentrations >50 nmol/L throughout and with only a few infants with <25 nmol/L (1%-5% depending on timepoint) (Table 5). Children HIV-unexposed uninfected had significantly poorer vitamin D status at WK14 (*p* < .001), with mean value <50 nmol/L and 22% of infants with <25 nmol/L. The group difference in vitamin D status was diminished at WK26, but still evident, and was no longer significant at later timepoints. This difference in pattern over time was significant (*p* < .0001 for interaction, Table 6).

**Table 5:** Biochemical markers of Gumba infants by group from 3 mo of age.

	**WK14**		**WK26**		**WK52**		**WK78**	
	**CHEU (*n* = 74)**	**CHUU (*n* = 74)**	**CHEU (*n* = 71)**	**CHUU (*n* = 72)**	**CHEU (*n* = 68)**	**CHUU (*n* = 62)**	**CHEU (*n* = 53)**	**CHUU (*n* = 60)**
**Vitamin D status**								
** 25OHD nmol/L**	70.0 (25.5)^a^	44.4 (24.0)	77.8 (23.3)^a^	63.6 (25.4)	70.5 (16.5)	68.7 (18.3)	68.6 (14.3)	68.1 (13.3)
**Hormones**								
** IGF1 μg/L**	52.6 (44.5, 64.2)	55.8 (47.0, 69.2)	44.2 (32.2, 60.4)	49.0 (37.0, 64.7)	42.3 (31.8, 61.7)	44.4 (34.0, 57.2)	47.0 (33.0, 63.4)	53.7 (35.0, 73.1)
** IGFBP3 μg/L**	2194 (1947, 2565)	2292 (1999, 2731)	2146 (1858, 2648)^c^	2393 (2133, 2765)	2230 (1919, 2709)	2312 (1959, 2781)	2260 (1886, 2702)	2472 (2206, 2858)
** IGF1/IGFBP3 molar**	0.089	0.090	0.076	0.076	0.070	0.071	0.077	0.081
	(0.077, 0.104)	(0.079, 0.105)	(0.064, 0.093)	(0.059, 0.096)	(0.058, 0.089))	(0.055, 0.096)	(0.062, 0.093)	(0.063, 0.105)
**Bone turnover markers**								
** P1NP μg/L**	2669 (2181, 3347)	2832 (2325, 3281)	1865 (1527, 2406)	1906 (1629, 2420)	1179 (954, 1592)	1134 (993, 1435)	1152 (964, 1505)	948 (840, 1176)
** CTX ng/L**	212 (162, 354)^a^	131 (81, 212)	472 (365, 759)	473 (307, 813)	605 (470, 1041)	565 (351, 926)	330 (213, 573)^c^	210 (118, 413)
** P1NP/CTX μg/ng**	12.6 (7.3, 17.8)^a^	21.7 (13.2, 33.6)	3.95 (2.50, 5.79)	4.03 (2.37, 5.87)	1.95 (1.18, 2.74)	2.01 (1.25, 2.93)	3.49 (1.89, 5.81)	4.51 (2.26, 7.97)
** OC μg/L**	45.6 (29.4, 74.1)	53.7 (38.1, 85.7)	65.5 (48.5, 97.0)	71.6 (49.7, 103.3)	60.3 (45.5, 85.1)	58.4 (42.8, 91.1)	59.8 (40.1, 92.2)	56.2 (42.3, 81.3)

Data for 25OHD are presented as means (SD). Data for hormones and bone turnover markers are expressed as geometric means (25th and 75th percentiles) obtained by back transformation from natural logarithms. Data are for infants who provided blood samples on ≥2 occasions *n* = 153 (CHEU 78, CHUU 75); infants with 4 samples = 62% (CHEU 55%, CHUU 68%), 3 samples = 26% (CHEU 31%, CHUU 21%), and 2 samples 12% (CHEU 14%, CHUU 11%).

Missing datapoints for 25OHD due to insufficient volume: WK14 CHEU = 1, WK26 CHUU = 1; WK52 CHEU = 2; WK 78 CHEU = 2, CHUU *n* = 1. There are no other analytes with missing data. Significance of difference between groups at each timepoint: ^a^*p* ≤ .001, ^b^*p* ≤ .01, ^c^*p* ≤ .05 from pairwise Scheffé post-hoc tests obtained in hierarchical 4-timepoint repeated measures models with the dependent variable converted to natural logarithms and with the independent variables timepoint, group, infant ID nested by group and a group*timepoint interaction term. 25OHD was modeled without logarithmic transformation. Differences between groups at each timepoint are summarized in Table 6. Within-individual percentage changes between timepoints in each group from the same models are summarized in Supplement S8.

Abbreviations: CHEU, Children HIV-exposed uninfected; CHUU, children HIV-unexposed uninfected; CTX, C-terminal telopeptide; IGF1, insulin-like growth factor 1; IGFBP3, insulin-like growth factor binding protein 3; IGF1/IGFBP3 molar ratio (to convert from μg/μg multiply IGF1 by 0.13 and IGFBP3 by 0.035); OC, osteocalcin; P1NP, procollagen type 1 N-terminal-propeptide; WK14, WK26, WK52, and WK78, 14, 26, 52, and 78 wk of age, respectively.

**Table 6:** Differences between CHEU and CHUU at each timepoint: biochemical factors.

	**WK2**	**WK14**	**WK26**	**WK52**	**WK78**	***p* for interaction**	***p* for group**	***p* for timepoint**
**Vitamin D status**								
**25OHD, nmol/L**	-	+25.4 (5.1)^a^	+12.9 (5.2)^a^	+0.8 (5.6)	−1.3 (6.2)	<.0001	.001	<.0001
**Hormones**								
**IGF1, %**	-	−5.8 (12.3)	−7.7 (12.6)	−5.9 (13.5)	−14.8 (14.7)	.79	.11	.0001
**IGFBP3, %**	-	−4.5 (6.2)	−9.0 (6.3)^**c**^	−2.0 (6.8)	−9.6 (7.4)	.34	.06	.72
**IGF1/IGFBP3, %**	-	−1.3 (8.3)	+1.3 (8.5)	−3.9 (9.1)	−5.2 (9.9)	.77	.59	<.0001
**Bone turnover markers**								
**P1NP, %**	-	−6.5 (13.5)	−0.5 (13.8)	+1.7 (14.8)	+15.8 (16.1)	.22	.57	<.0001
**CTX, %**	-	+50.9 (24.1)^a^	−0.1 (24.7)	+3.2 (26.4)	+45.6 (28.9)^**c**^	.004	.002	<.0001
**P1NP/CTX, %**	-	−57.4 (24.2)^a^	+0.5 (24.7)	−1.5 (26.5)	−29.8 (29.0)	.003	.01	<.0001
**OC, %**	-	−17.8 (17.1)	−8.5 (17.5)	+1.8 (18.7)	+3.5 (20.5)	.34	.41	<.0001

Data are mean percentage differences (95% CI) from pairwise Scheffé post-hoc tests obtained in hierarchical models with timepoint, group, infant ID nested in group and a timepoint^*^group interaction term with data transformed to natural logarithms. 25OHD was modeled without logarithmic transformation and differences are in nmol/L. For models where the interaction term was not significant, removing the interaction term made no material effect to the *p*-values for group or timepoint. Data are for infants who were measured on ≥2 occasions, the values and numbers are given in Table 5.

Difference between groups at each timepoint ^a^*p* ≤ .001, ^b^*p* ≤ .01, ^c^*p* ≤ .05. Within-individual changes between timepoints from the same models are summarized in Supplement S8.

Abbreviations: CHEU, children HIV-exposed uninfected; CHUU, children HIV-unexposed uninfected; CTX, C-terminal telopeptide; IGF1, insulin-like growth factor 1; IGFBP3, insulin-like growth factor binding protein 3; IGF1/IGFBP3, IGF1/IGFBP3 molar ratio; OC, osteocalcin; P1NP, procollagen type 1 N-terminal-propeptide; WK2, WK14, WK26, WK52, and WK78, 2, 14, 26, 52, and 78 wk of age, respectively.

**Figure 2:**
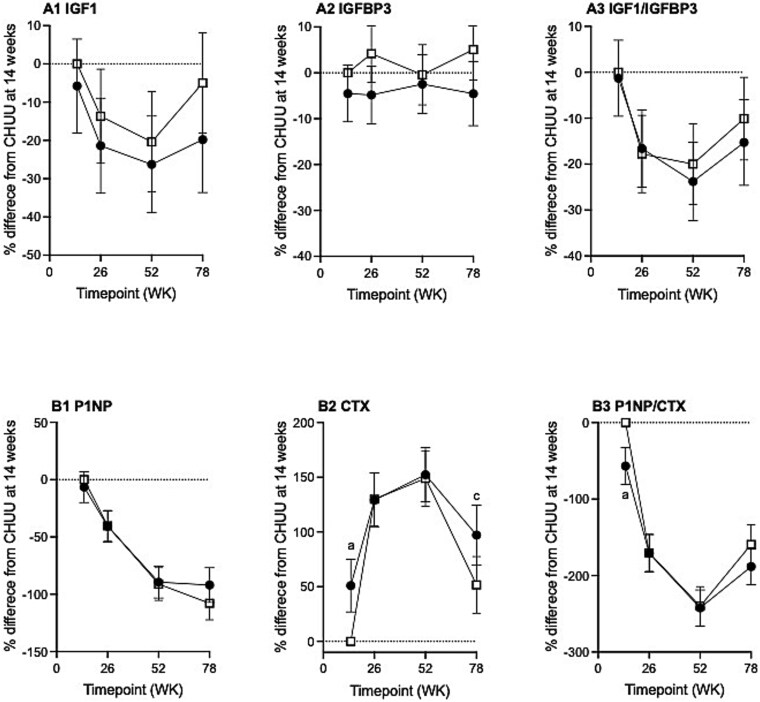
Biochemical markers of Gumba infants. Percent changes during infancy from age 14 wk expressed as a difference from CHUU at WK14. Data are percentage means ±95% CIs obtained from pairwise Scheffé post-hoc tests for group^*^timepoint interaction terms in hierarchical repeated measures ANOVA models of data converted to natural logarithms with timepoint, group, infant ID nested within group and timepoint^*^group interaction of data converted to natural logarithms. Symbols: black rounds, children HIV-exposed uninfected (CHEU); white squares, children HIV-unexposed uninfected (CHUU). The numbers of children measured at each timepoint can be found in Table 5, the differences between groups at each timepoint in Table 6 and the within-individual changes by group in Supplement S8. Panel (A1-3) Growth markers at each timepoint by group: IGF1, insulin-like growth factor 1; IGFBP3, insulin-like growth factor binding protein 3; IGF1/IGFBP3, molar ratio of IGF1 to IGFBP3. Panel (B1-3) Bone turnover markers at each timepoint by group: P1NP, procollagen type 1 N-terminal-propeptide; CTX, C-terminal telopeptide; P1NP/CTX, ratio of P1NP to CTX. Significance of difference between groups a, *p* ≤ .001; b, *p* ≤ .01; c, *p* ≤ .05.

The patterns of change over time in the growth factors and bone turnover markers are illustrated in Figure 2. Concentrations of IGF1 decreased from WK14 to WK26 by around 15% and increased after WK52 (Figure 2A1). The concentrations of IGFBP3 did not change significantly over time (Table 6, Figure 2A2). As a consequence, changes in the ratio of IGF1/IGFBP3 paralleled those of IGF1, decreasing after WK14 and rising after WK52 (Figure 2A3). There were no significant group differences in the growth factors at any timepoint except for IGFBP3 at WK26 when CHEU had lower values (Table 6).

There were no differences evident in P1NP between the groups. P1NP concentrations decreased rapidly from WK14 to WK78, a fall of around 100% in both groups. In contrast, CHEU had higher concentrations of CTX than CHUU at WK14 by 51% (*p* < .001, Table 6). CTX concentrations rose rapidly in both groups after WK14 and no difference between the groups was evident at WK26 and WK52. CTX concentrations fell after WK52 and the significant group difference re-emerged at WK78 (46%, *p* = .02, Table 6). The difference in the pattern of change in CTX concentrations between the groups was significant (*p* = .004 for interaction, Table 6). Similarly, the pattern of changes in P1NP/CTX was an approximate inverse of that of CTX and was different in the 2 groups (*p* = .003 for interaction, Table 6). There were no group differences in OC, which increased in concentration from WK14 to WK26 and then decreased in both groups (Table 6, Supplement S8).

Females tended to have higher IGF1 and IGF1/IGFBP3 than males, the differences increasing with age. Adjusting for sex did not alter the lack of a group difference in either IGF1 or IGF1/IGFBP3. There was also no evidence of an association between weight and length with IGF1 at any timepoint or in increases over time in models with or without sex adjustment. CTX was higher in first-born infants, but adjustment for maternal parity did not affect the group differences in the longitudinal models. There were no sex or maternal parity effects noted among the other biochemical factors, nor was there any evidence of effects of exclusive breastfeeding or maternal prior use of DMPA on any of the measured biochemical factors.

The sensitivity analyses for the anthropometry and LS bone measures confirmed and strengthened the significance of group differences seen with the full dataset (Supplements S9 and S10). Those for WB bone measures, body composition, and biochemical data demonstrated similar patterns to the full dataset but strengths of significance were diminished (Supplements S9 and S11, respectively).

## Discussion

This paper presents detailed longitudinal data on somatic and bone growth in a well-profiled cohort of breastfed Ugandan CHEU, with cumulative in utero and postpartum exposure to TDF-based maternal ART, compared to CHUU peers. Infants in both groups had similar weight and length at 2 wk of age. However, CHEU had slower growth by 3 mo such that both body weight and fat mass were about 0.5 kg lower by 6 mo compared to CHUU. Both groups experienced growth faltering from 6 mo of age relative to international reference data with the divergence in weight and length increasing over time, such that by 78 wk of age 59% of CHEU were classified as stunted compared to 33% of CHUU. Rates of stunting in CHUU at 18 mo were comparable to the national prevalence reported for children aged 12-23 mo in 2016.^34^ Children HIV-exposed uninfected also had lower WB BMC and BA at 14 and 26 wk of age, and LS BMC and BA were lower at 2 and 14 wk. There were no group differences in WB or LS aBMD before or after size-adjustment, indicating that the lower bone mineral accretion in CHEU was appropriate for their smaller bone and body size. Overall, these data show the onset of somatic and skeletal growth deficits in CHEU during the first 3 mo of life, setting them on a lower growth trajectory compared to CHUU peers.

Growth deficits in CHEU are well recognised^4^^,^^5^^,^^35^ and lower WB BMC has been reported in neonates exposed to maternal TDF in utero.^15^ The PROMISE 1077 BFtrial/1084s substudy trial, compared the effects on infant bone in African CHEU exposed to maternal TDF-based ART postpartum vs CHEU on nevirapine prophylaxis.^36^ They reported slower LS bone mineral accretion between 6-21 d and 26 wk of age, with significantly lower LS BMC at 26 wk, in CHEU exposed to TDF through breastmilk compared to CHEU treated with nevirapine.^14^ The difference in LS BMC reported in PROMISE 1084s after 26 wk exposed to TDF was −0.45 SD-score, which was similar to that seen in Gumba CHEU at 2 wk in infants whose mothers had initiated ART in pregnancy approximately 18 wk previously (−0.48 SD-score).

Several underlying factors have been considered in the literature as possible drivers of the growth deficits in CHEU. These included exposure to maternal HIV and ART, co-infections and immune activation, nutrition, vitamin D deficiency, socio-economic, and environmental factors.^6^^,^^37^^,^^38^ The specific interest in maternal TDF exposure relates to the known bone and renal effects in adults.^7–9^^,^^37^ There were relatively few differences in the dietary and biochemical data from the Gumba infants to account for the differences in somatic and bone growth between CHEU and their CHUU peers. In addition, there were relatively few differences relating to the family environment and maternal circumstances that could account for these differences. Fewer of the mothers of CHEU were primiparous, married, and educated to post-secondary level and had access to piped drinking water than mothers of CHUU, but otherwise the health, demographic, and socioeconomic status of the mothers were comparable.^9^^,^^39^ The majority of infants in both groups were breastfed to at least 6 mo, with higher rates of exclusive breastfeeding among CHEU, but all CHEU had stopped breastfeeding by 18 mo. Diet adequacy was poor in both groups of infants after 6 mo, with little to distinguish between them in the quality of their family diets at 52 and 18 mo. Vitamin D status of the CHEU infants was good at all ages, while those of CHUU was poorer during early infancy. All CHEU were on cotrimoxazole prophylaxis, had fewer episodes of illness, and a greater proportion were exclusively breastfed in the first 6 mo of life compared to CHUU which could have disposed towards the null. In this resource-poor community, children who were exclusive breastfed had greater weight, fat mass, and bone accretion in the first 6 mo compared to those who were mixed-fed. Furthermore, cotrimoxazole use has been associated with improved growth outcomes in HIV-infected children.^40^^,^^41^ Hence, the early onset of growth deficits in CHEU in the current study is unlikely to have been driven by differences in maternal circumstances or rates of exclusive breastfeeding, poor diet quality, vitamin D deficiency or acute illnesses in the first 3 mo of life. Similar growth deficits have also been reported in Nigerian CHEU with cumulative in utero and postpartum maternal ART exposure despite higher rates of exclusive breastfeeding compared to CHUU peers.^35^

There were also no significant differences noted in the biochemical growth factors or in the bone formation markers P1NP and OC. In Malawian CHEU with cumulative in utero and postpartum maternal ART exposure, the bone formation marker bone specific alkaline phosphatase was also considerably higher compared to European and Asian thresholds at 6 and 12 mo, with no significant difference by maternal TDF exposure.^16^ Notably, in the Gumba CHEU, the bone resorption marker CTX was significantly higher than CHUU at 14 wk and at 78 wk, although, as in the Malawian study,^16^ not at 6 and 12 mo when both groups had raised concentrations. Maternal bone biomarkers have been detected in breastmilk^42^^,^^43^ and crosslinked N-terminal of telopeptide 1 collagen (a marker of bone resorption, related to CTX) is higher in exclusively breastfed infants.^43^ CTX was higher in the mothers of Gumba CHEU infants,^9^^,^^17^^,^^18^ possibly linked to the TDF-containing ART initiated during pregnancy. It is plausible, therefore, that altered bone metabolism could be one of the drivers for observed early growth deficits in CHEU infants, and that their higher concentrations of CTX were either of maternal origin via breastmilk or a direct effect of in-utero or postpartum exposure to TDF on infant bone metabolism.

The current study has several strengths. First, the data are from a well-characterized prospective African cohort of a substantial number of mother-infant pairs with known maternal bone outcomes and where CHEU were exposed to a single maternal triple ART regimen, TDF-3TC-EFV.^9^^,^^17^^,^^18^ A comparative group was included of CHUU born to the HIV-negative REF mothers in the cohort, thereby providing reference data not only for Gumba CHEU but also for the general population of local infants. Second, anthropometry, good quality infant WB, and LS DXA scans and blood samples for markers of growth and bone metabolism were obtained at several timepoints across the first 18 mo of life, providing a comprehensive picture of growth trajectories. In addition, besides WHO growth Z-scores, we report all WB and LS DXA bone measures (BMC, BA, and aBMD) and body composition (lean and fat mass). To date, only a few studies have reported DXA and biochemical data in African children with^14–16^ and without^44^ exposure to maternal HIV, ART, or TDF, and studies in CHEU did not include a comparative group of CHUU.

However, there are limitations to the study. This was an observational cohort study and it is not possible to determine whether the differences between groups were due to disparities in education and socioeconomic status of mothers^39^ or to unmeasured factors such as maternal infection burden and nutrition during pregnancy.^6^^,^^37^^,^^38^ Since all CHEU in the Gumba study were exposed to a single maternal ART regimen, it is not possible to disentangle the effects of HIV, TDF, and other ART, either individually or in combination, on the observed growth and skeletal outcomes. Although the sample size was sufficient to consider differences in anthropometry and bone measures, it may have been too small to uncover subtle differences in the biochemical factors because of their greater between- and within-individual variation. Further, although rates of exclusive breastfeeding in the first 6 mo were higher in CHEU, their rates of continued breastfeeding in the second year or life were lower. This was in accord with the advice given to mothers living with HIV at the time to discontinue breast-feeding after 12 mo.^19^^,^^20^ It is therefore not possible to know whether there were differences in breastmilk volume and/or components that may have influenced the growth and bone outcomes. In addition, we only assessed diet quality not quantitative dietary intake. Lastly, DXA scans were acquired only up to 6 mo of life, and it is therefore not possible to determine whether the attained bone mineral was adequate for body size at older ages.

In conclusion, the current study demonstrated slower somatic and skeletal growth and altered bone metabolism by 3 mo of life in breastfed Ugandan CHEU with cumulative in utero and postpartum maternal TDF-based ART. These coincided with accentuated bone mineral mobilization and disruptions in calcium and bone metabolism in their mothers.^9^^,^^17^^,^^18^ Poor growth in early life, especially in the first 1000 d of life (from conception to 2 yr postpartum), is associated with long-term adverse consequences, including impaired cognitive development, that persist into adulthood.^45^ Further, persistent poor growth has been reported in Uganda CHEU at 60 mo.^5^ As the population of African WLH and CHEU with antepartum and postpartum exposure to maternal HIV/ART continues to grow,^13^ tracking CHEU to deliver interventions after discharge from EID follow-up at 18 mo may not be feasible because of practical, economic and privacy challenges. Longitudinal studies of mother-infant dyads including measures of breastmilk volume and composition, with a special focus on pregnancy and the first 6 mo of life, are, therefore, urgently needed to inform strategies to promote growth in African CHEU in the first 1000 d of life under the current universal ART guidelines.

## Supplementary Material

Supplementary_materials_zjag024

## Data Availability

Data from the Gumba Study are archived with UKRI-MRC. The procedure for requesting data access can be found at https://www.ukri.org/publications/access-to-data-from-the-mrc-science-archive-application-form/.
