# Supplementary material for: Longitudinal comparison of bone, growth, and biochemical markers in breastfed HIV-exposed uninfected and HIV-unexposed Ugandan children
Source: J Bone Miner Res. 2026 Feb 3;41(8):823–36. doi: 10.1093/jbmr/zjag024 (PMC13421069; doi:10.1093/jbmr/zjag024)
Supplement: Supplementary_materials_zjag024 [file supplementary_materials_zjag024.docx]

## **Supplement S1. Biological samples: collection, processing and laboratory analysis**

At WK14, WK26, WK52 and WK78, non-fasted venous blood (2 ml) was collected from the arm into plain serum tubes, following prevailing standard laboratory procedures for blood collection in children at the Baylor-Uganda Clinical Center of Excellence for Pediatric HIV. All blood samples were centrifuged, aliquoted and stored at -80^0^C in Uganda and later airfreighted on dry ice, in batches, for storage and laboratory analysis at the MRC Elsie Widdowson Laboratory and the MRC Epidemiology Unit in Cambridge, UK.

Serum samples were analysed for vitamin D status (25-hydroxyvitamin D, 25OHD), hormones involved in growth and bone metabolism: intact insulin-like growth factor 1 (IGF1), IGF-binding protein 3 (IGFBP3) and bone turnover markers procollagen type 1 N-terminal-propeptide (P1NP), β-C-terminal telopeptide (CTX) and osteocalcin (OC). Total 25OHD was assayed by isotope dilution ultra performance liquid chromatography-tandem mass spectrometry^1^ and the growth factors and bone turnover markers were assayed using the IDS iSyS platform and kits (Immunodiagnostics Systems Ltd, Tyne and Wear, UK). The index IGF1/IGFBP3 is used as a marker of growth hormone deficits and is presented as a molar ratio obtained from data in µg/L by multiplying IGF1 by 0.13 and IGFBP3 by 0.035.^2,3^ P1NP is regarded as a bone formation marker, CTX as a bone resorption marker, and circulating OC as a mix of osteoblastic factors released during formation and related fragments released during resorption. The ratio of P1NP/CTX is used as an index of the balance between bone formation and bone resorption.^4^

At the time of these measurements, the laboratory participated in the international Vitamin D External Quality Assessment Scheme (DEQAS, www.deqas.org) and held a certificate of proficiency for 25OHD. For the other analytes, standards supplied by the manufacturers were used for quality assurance. P1NP concentrations in the infants were above the assay range of the IDS kit and were re-assayed diluted with 5% bovine serum albumin in phosphate-buffered saline. CTX concentrations in seven samples were below the limit of detection (LOD) of the IDS kit (0.033 ng/L) and were included in the dataset as LOD divided by the square root of 2 (0.023 ng/L).

References

1. Jones KS, Meadows SR, Schoenmakers I, Prentice A, Moore SE. Vitamin D status increases during pregnancy and in response to vitamin D supplementation in rural Gambian women. J Nutr. 2020;150(3):492–504.

2. Wu W, Gong C, Li Y, et al. Long-term efficacy and safety of recombinant human growth hormone in children born small for gestational age. Horm Metab Res. 2023/09/07 2023;55(09):599-609. doi:10.1055/a-2136-8654

3. Laboratories MC. Insulin-Like Growth Factor 1 and Insulin-Like Growth Factor-Binding Protein 3 Growth Panel, Serum. Mayo Clinic Laboratories. Accessed 07-04-2025, 2025. <https://www.mayocliniclabs.com/test-catalog/overview/36365#Clinical-and-Interpretive>

4. Christodoulou M, Aspray TJ, Piec I, et al. Vitamin D supplementation for 12 months in older adults alters regulators of bone metabolism but does not change Wnt signaling pathway markers. JBMR Plus 2022;6:e10619.

**Supplement S2: Chart of participant flow through the study**


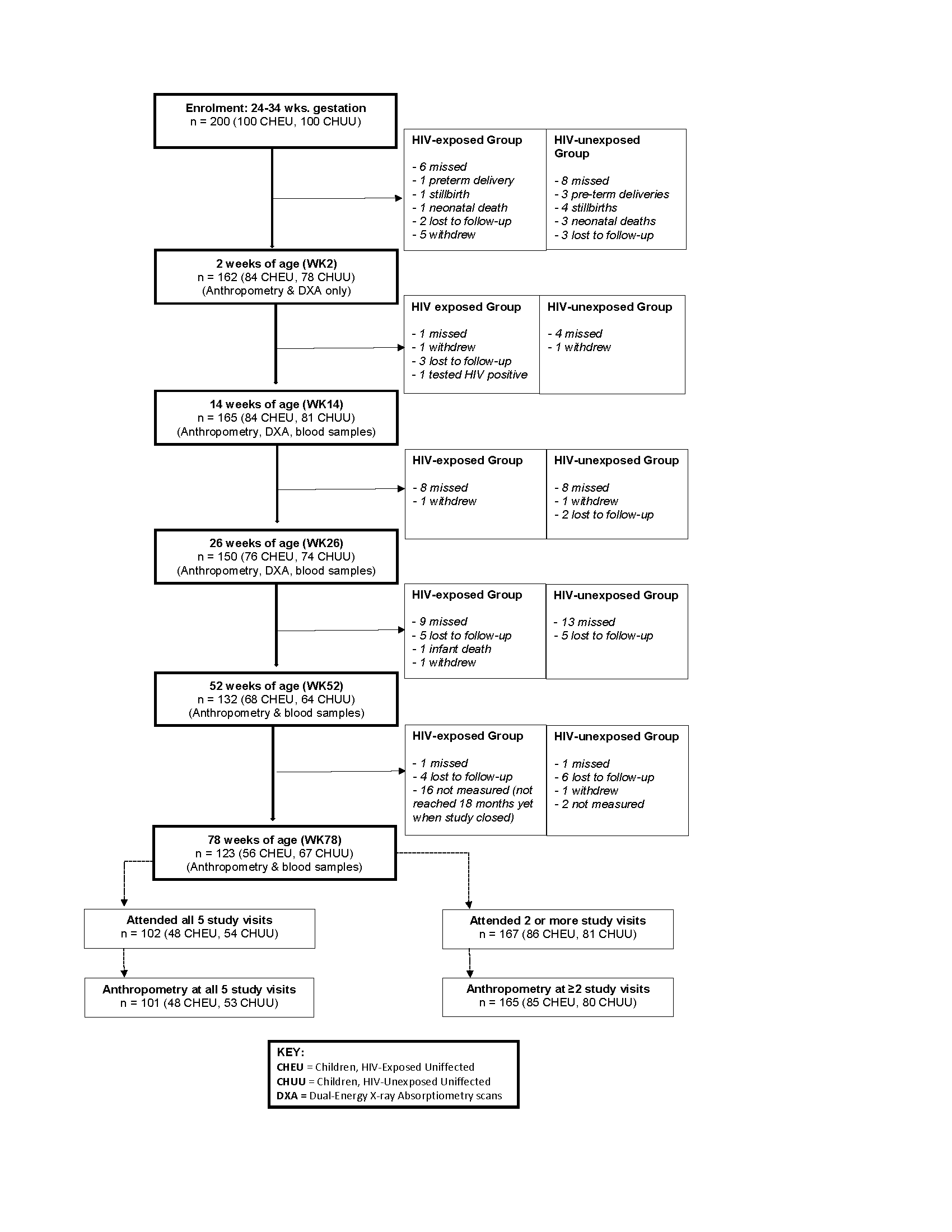


**Supplement S3: Summary of measurements and numbers included in final hierarchical models by group and timepoint.**

|  | WK2 | | | | |  | | WK14 | | | | |  | | | WK26 | | | | | |  | | WK52 | | | |  | | | WK78 | | | |  |  |
| --- | --- | --- | --- | --- | --- | --- | --- | --- | --- | --- | --- | --- | --- | --- | --- | --- | --- | --- | --- | --- | --- | --- | --- | --- | --- | --- | --- | --- | --- | --- | --- | --- | --- | --- | --- | --- |
|  | CHEU | CHUU | | | | |  | | CHEU | CHUU | | | | |  | | CHEU | | CHUU | |  | | CHEU | | | CHUU | | |  | | | CHEU | | CHUU | |  |
| *Anthropometry* | |  | |  |  |  | | | | | |  |  | |  | | | |  | |  | |  | | |  | |  | | |  | | |  | |  |
| Weight | | 81 | | 75 |  | 83 | | | | | | 79 |  | | 75 | | | | 73 | |  | | 68 | | | 64 | |  | | | 56 | | | 66 | |  |
| Length | | 81 | | 75 |  | 83 | | | | | | 79 |  | | 75 | | | | 73 | |  | | 67 | | | 64 | |  | | | 56 | | | 66 | |  |
| MUAC | | - | | - |  | - | | | | | | - |  | | 75 | | | | 71 | |  | | 68 | | | 64 | |  | | | 56 | | | 66 | |  |
| HC | | 80 | | 72 |  | 83 | | | | | | 79 |  | | 75 | | | | 72 | |  | | 68 | | | 64 | |  | | | 56 | | | 65 | | |
| WAZ | | 81 | | 75 |  | 83 | | | | | | 79 |  | | 75 | | | | 73 | |  | | 67 | | | 64 | |  | | | 54 | | | 64 | |  |
| LAZ | | 81 | | 75 |  | 83 | | | | | | 79 |  | | 75 | | | | 73 | |  | | 66 | | | 64 | |  | | | 54 | | | 64 | |  |
| WFLZ | | 81 | | 75 |  | 83 | | | | | | 79 |  | | 75 | | | | 73 | |  | | 66 | | | 64 | |  | | | 54 | | | 64 | |  |
| MAZ | | - | | - |  | - | | | | | | - |  | | 75 | | | | 71 | |  | | 67 | | | 64 | |  | | | 54 | | | 64 | |  |
| HCAZ | | 80 | | 72 |  | 83 | | | | | | 79 |  | | 75 | | | | 72 | |  | | 67 | | | 64 | |  | | | 54 | | | 63 | | |
| *DXA measures* | |  | |  |  |  | | | | | |  |  | |  | | | |  | |  | |  | | |  | |  | | |  | | |  | |  |
| Whole body | | 69 | | 69 |  | 76 | | | | | | 75 |  | | 69 | | | | 69 | |  | | - | | | - | |  | | | - | | | - | |  |
| Lumbar spine | | 77 | | 74 |  | 80 | | | | | | 79 |  | | 65 | | | | 69 | |  | | - | | | - | |  | | | - | | | - | |  |
| Body comp’n | | 69 | | 67 |  | 76 | | | | | | 75 |  | | 69 | | | | 69 | |  | | - | | | - | |  | | | - | | | - | |  |
| *Biochemistry* | |  | |  |  |  | | | | | |  |  | |  | | | |  | |  | |  | | |  | |  | | |  | | |  | |  |
| 25OHD | | - | | - |  | 73 | | | | | | 74 |  | | 71 | | | | 71 | |  | | 66 | | | 62 | |  | | | 51 | | | 59 | |  |
| IGF1 | | - | | - |  | 74 | | | | | | 74 |  | | 71 | | | | 72 | |  | | 68 | | | 62 | |  | | | 53 | | | 60 | |  |
| IGFBP3 | | - | | - |  | 74 | | | | | | 74 |  | | 71 | | | | 72 | |  | | 68 | | | 62 | |  | | | 53 | | | 60 | |  |
| P1NP | | - | | - |  | 74 | | | | | | 74 |  | | 71 | | | | 72 | |  | | 68 | | | 62 | |  | | | 53 | | | 60 | |  |
| CTX | | - | | - |  | 74 | | | | | | 74 |  | | 71 | | | | 72 | |  | | 68 | | | 62 | |  | | | 53 | | | 60 | |  |
| Osteocalcin | | - | | - |  | 74 | | | | | | 74 |  | | 71 | | | | 72 | |  | | 68 | | | 62 | |  | | | 53 | | | 60 | |  |

Footnote to Supplement S3

Values are number of measurements or samples analysed for each analyte; missing data points were due to missing visits or poor-quality measures for anthropometry and DXA, or to insufficient/no sample for biochemistry. Data are for infants who were measured on ≥2 occasions in each category.

Abbreviations: 25OHD = 25-hydroxyvitamin D, CHEU =Children HIV-exposed uninfected, CHUU = children HIV-unexposed uninfected, CTX = C-terminal telopeptide, HC = head circumference, HCAZ = head circumference-for-age SD score, IGF1 = insulin-like growth factor 1, IGFBP3 = insulin-like growth factor binding protein 3, LAZ = length-for-age SD-score, MUAC = mid-upper arm circumference. MAZ = MUAC-for-age SD score, OC = osteocalcin, P1NP = procollagen type 1 N-terminal-propeptide, WAZ = weight-for-age SD-score, WFLZ = weight-for-length SD-score, WK2, WK14, WK26, WK52 and WK78 = 2, 14, 26, 52 and 78 weeks of age, respectively.

**Supplement S4: Feeding practices of Gumba infants at 12 and 18 months, qualitative 24 hour diet recall**

|  | WK52 | | |  | WK 78 | | |  |
| --- | --- | --- | --- | --- | --- | --- | --- | --- |
|  | CHEU  (n=66) | CHUU  (n=63) | *P* |  | CHEU  (n=54) | CHUU  (n=65) | *P* | |
| Breastfed/breastmilk (%yes) | 60.3 | 88.9 | 0.0002 |  | 0.0 | 43.3 | <0.0001 | |
| Non-breastfed children, ≥2 animal milk feeds (%yes) | 25.9 | 28.2 | 0.89 |  | 22.2 | 25.0 | 0.76 | |
| Grains/roots/tubers (%yes) | 98.5 | 95.2 | 0.29 |  | 98.1 | 98.5 | 0.89 | |
| Legumes/nuts (%yes) | 56.1 | 47.6 | 0.38 |  | 74.1 | 64.6 | 0.27 | |
| Milk and dairy products (%yes) | 78.8 | 65.1 | 0.08 |  | 68.5 | 70.8 | 0.79 | |
| Flesh foods (%yes) | 41.5 | 49.2 | 0.44 |  | 35.2 | 43.1 | 0.38 | |
| Eggs (%yes) | 18.2 | 23.8 | 0.43 |  | 20.4 | 27.7 | 0.35 | |
| Vit A rich fruits and vegetables (%yes) | 10.6 | 11.1 | 0.93 |  | 11.1 | 10.8 | 0.95 | |
| Other fruits and vegetables (%yes) | 36.4 | 38.1 | 0.84 |  | 33.3 | 41.5 | 0.36 | |
| Minimum DD score, MDD (%yes) | 28.8 | 33.3 | 0.56 |  | 11.1 | 27.7 | 0.025 | |
| Minimum Meal Frequency, MMF (%yes) | 71.2 | 76.2 | 0.52 |  | 29.6 | 55.4 | 0.005 | |
| Minimum Acceptable Diet, MAD (%yes) | 22.7 | 33.3 | 0.18 |  | 5.6 | 23.1 | 0.008 | |

Data are for infants who were measured on ≥2 occasions with 24-hour qualitative dietary intake data. Values are percentages of children who had consumed the food (any amount) in the past 24 hours or received a diet that met the WHO recommendations for minimum dietary diversity (MDD), meal frequency (MMF), overall diet quality (MAD), and milk feeds for non-breastfed children aged 12-23 months. Abbreviations: CHEU = children HIV-exposed uninfected, CHUU = children HIV-unexposed uninfected children. Significance of difference between groups at each timepoint (*P*-values) are from chi-square tests obtained in two-by-two contingency tables.

Supplement S5: DXA local SD-Scores for CHEU infants at each timepoint

|  | WK2 | | WK14 | | WK26 | |
| --- | --- | --- | --- | --- | --- | --- |
| *WB Bone measures* |  | |  | |  | |
| BMC SDS | +0.01 (0.20) | | -0.34 (0.21)**^b^** | | -0.40 (0.22)**^a^** | |
| BA SDS | +0.05 (0.20) | | -0.40 (0.21)**^a^** | | -0.41 (0.21)**^a^** | |
| aBMD SDS | +0.01 (0.20) | -0.18 (0.22) | | -0.09 (0.25) | |  |
| *LS Bone measures* |  | |  | |  | |
| BMC SDS | -0.46 (0.22)**^a^** | | +0.17 (0.23) | | -0.13 (0.25) | |
| BA SDS | -0.51 (0.25)**^a^** | | -0.58 (0.26)**^a^** | | -0.24 (0.30) | |
| aBMD SDS | -0.23 (0.25) | | +0.13 (0.21) | | +0.01 (0.22) | |
| *Body composition* |  | |  | |  | |
| Lean mass SDS | -0.04 (0.20) | | -0.27 (0.17)^b^ | | +0.01 (0.22) | |
| Fat mass SDS | -0.05 (0.20) | | -0.16 (0.21) | | -0.38 (0.22)^b^ | |
|  |  | |  | |  | |

Data are mean SD-scores (95% confidence intervals) in CHEU expressed relative to CHUU at each timepoint. Data are for infants who were scanned on ≥2 occasions in each category, the values and numbers are given in Table 3.

Abbreviations: aBMD = areal bone mineral density; BA =bone area; BMC = bone mineral content; CHEU = children HIV-exposed uninfected; CHUU = children HIV-unexposed uninfected; LS = lumbar spine L1-4; SDS = local standard deviation score; WB = whole-body with head; WK2, WK14, WK26 = 2, 14, 26, weeks of age, respectively.

**^a,b,c^** Significance of difference between groups at each timepoint by one sample two-tailed t-test **^a^***P* ≤0.001, **^b^***P* ≤0.01,

Supplement S6: Differences between CHEU and CHUU at each timepoint: anthropometric SD-scores

|  | WK2 | WK14 | WK26 | WK52 | WK78 | *P*interaction | *P*group | *P*timepoint |
| --- | --- | --- | --- | --- | --- | --- | --- | --- |
|  |  |  |  |  |  |  |  |  |
| WAZ | -0.17 (0.20) | -0.46 (0.19)**^a^** | -0.45 (0.20)**^a^** | -0.26 (0.22) | -0.38 (0.24)^c^ | 0.20 | 0.02 | <0.0001 |
| LAZ | -0.22 (0.26) | -0.56 (0.26)**^a^** | -0.35 (0.27) | -0.42 (0.30) | -0.43 (0.31) | 0.47 | 0.01 | <0.0001 |
| WFLZ | +0.02 (0.31) | -0.14 (0.30) | -0.33 (0.32) | -0.12 (0.35) | -0.28 (0.37) | 0.61 | 0.26 | <0.0001 |
| MAZ | - | - | -0.25 (0.19)**^c^** | -0.21 (0.20) | -0.17 (0.22) | 0.84 | 0.17 | 0.12 |
| HCAZ | -0.11 (0.28) | -0.24 (0.27) | -0.02 (0.29) | -0.22 (0.31) | -0.39 (0.33) | 0.52 | 0.20 | <0.0001 |

Data are mean differences (95% confidence intervals) from pairwise Scheffé post-hoc tests obtained in hierarchical models with timepoint, group, infant ID nested in group and a timepoint*group interaction term. For models where the interaction term was not significant, removing the interaction term made no material effect to the *P*-values for group or timepoint. Data are for infants who were measured on ≥2 occasions in each category, the values and numbers are given in Table 2.

Abbreviations: CHEU = children HIV-exposed uninfected; CHUU = children HIV-unexposed uninfected; HCAZ = head circumference-for-age SD-score; LAZ = length-for-age SD-score; MAZ = mid-upper arm circumference-for-age SD-score; WAZ = weight-for-age SD-score; WFLZ = weight-for-length SD-score; WK2, WK14, WK26, WK52, WK78 = 2, 14, 26, 52, 78 weeks of age, respectively.

**^a,b,c^** Significance of difference between groups at each timepoint **^a^***P* ≤0.001, **^b^***P* ≤0.01, **^c^***P* ≤0.05. Within-individual changes between timepoints from the same models are summarised in Supplement S7.

Supplement S7: Within-individual changes between timepoints in Gumba infants by group: anthropometry and DXA measures.

|  | WK2 to WK14 | | WK14 to WK26 | | | WK26 to WK52 | | | | WK52 to WK78 | | | |  |
| --- | --- | --- | --- | --- | --- | --- | --- | --- | --- | --- | --- | --- | --- | --- |
|  | CHEU | CHUU | CHEU | CHUU | | CHEU | | CHUU | | CHEU | | CHUU | |  |
| *Anthropometry* |  |  |  | |  | |  | |  | |  | |  | |
| Weight, kg | +2.49 (0.18)**^a^** | +2.78 (0.18)**^a^** | +1.25 (0.18)**^a^** | | +1.31 (0.19)**^a^** | | +1.41 (0.19)**^a^** | | +1.37 (0.20)**^a^** | | +0.96 (0.21)**^a^** | | +1.14 (0.20)**^a^** | |
| Length, cm | +8.98 (0.61)**^a^** | +9.73 (0.63)**^a^** | +5.30 (0.63)**^a^** | | +5.17 (0.64)**^a^** | | +5.22 (0.67)**^a^** | | +5.67 (0.68)**^a^** | | +4.77 (0.72)**^a^** | | +5.09 (0.69)**^a^** | |
| MUAC, cm | - | - | - | | - | | +0.57 (0.22)**^a^** | | +0.51 (0.23)**^a^** | | +0.34 (0.24)**^c^** | | +0.24 (0.23) | |
| HC, cm | +4.40 (0.31)**^a^** | +4.63 (0.32)**^a^** | +2.31 (0.32)**^a^** | | +2.26 (0.32)**^a^** | | +2.52 (0.33)**^a^** | | +2.66 (0.34)**^a^** | | +1.29 (0.36)**^a^** | | +1.50 (0.35)**^a^** | |
| WAZ | -0.02 (0.19) | +0.26 (0.20) | -0.10 (0.20) | | -0.10 (0.20) | | -0.18 (0.21) | | -0.37 (0.21)**^c^** | | -0.17 (0.23) | | -0.06 (0.22) | |
| LAZ | -0.05 (0.26) | +0.29 (0.26) | -0.04 (0.26) | | -0.26 (0.27) | | -1.10 (0.28)**^a^** | | -1.03 (0.28)**^a^** | | -0.37 (0.31) | | -0.35 (0.29) | |
| WFLZ | +0.35 (0.30) | +0.51 (0.31)**^c^** | -0.18 (0.31) | | +0.01 (0.32) | | +0.32 (0.33) | | +0.12 (0.34) | | -0.06 (0.36) | | +0.10 (0.34) | |
| MAZ | - | - | - | | - | | +0.13 (0.19) | | +0.09 (0.20) | | +0.06 (0.21) | | +0.02 (0.20) | |
| HCAZ | -0.24 (0.27) | -0.11 (0.28) | -0.14 (0.28) | | -0.35 (0.28) | | -0.10 (0.29) | | +0.10 (0.30) | | -0.04 (0.32) | | +0.13 (0.31) | |
| *Bone measures* |  |  |  | |  | |  | |  | |  | |  | |
| WB BMC, g | +53.0 (3.7)**^a^** | +59.0 (3.6)**^a^** | +35.9 (3.7)**^a^** | | +36.1 (3.6)**^a^** | | - | | - | | - | | - | |
| WB BA, cm^2^ | +188.7 (10.2)**^a^** | +213.5 (10.2)**^a^** | +95.1 (10.3)**^a^** | | +98.6 (10.2)**^a^** | | - | | - | | - | | - | |
| WB aBMD, g/cm^2^ | +0.028(0.004)**^a^** | +0.030(0.004)**^a^** | +0.022(0.004)**^a^** | | +0.020(0.004)**^a^** | | - | | - | | - | | - | |
| LS BMC, g | +0.53 (0.08)**^a^** | +0.46 (0.08)**^a^** | +0.53 (0.08)**^a^** | | +0.54 (0.08)**^a^** | | - | | - | | - | | - | |
| LS BA, cm^2^ | +2.41 (0.23)**^a^** | +2.55 (0.23)**^a^** | +1.42 (0.24)**^a^** | | +1.17 (0.24)**^a^** | | - | | - | | - | | - | |
| LS aBMD, g/cm^2^ | +0.004 (0.006) | -0.006 (0.006) | +0.020 (0.006)**^a^** | | +0.024 (0.006)**^a^** | | - | | - | | - | | - | |
| *Body Composition* |  |  |  | |  | |  | |  | |  | |  | |
| Lean mass, g | +627 (200)**^a^** | +829 (201)**^a^** | +984 (201)**^a^** | | +747 (199)**^a^** | | - | | - | | - | | *-* | |
| Fat mass, g | +1892 (235)**^a^** | +2048 (237)**^a^** | +322 (236)**^a^** | | +632 (234)**^a^** | | - | | - | | - | | *-* | |
| %Fat | +18.3 (3.0)**^a^** | +18.6 (3.0)**^a^** | -3.5 (3.0) | | +0.2 (3.0) | | - | | - | | - | | *-* | |

Footnote to Supplement S7.

Data are mean differences (95% confidence intervals) from pairwise Scheffé post-hoc tests obtained in hierarchical models with timepoint, group, infant ID nested in group and a timepoint*group interaction term. Data are for infants who were measured on ≥2 occasions, the values and numbers are given in Tables 2 and 3.

Abbreviations: aBMD = areal bone mineral density; BA =bone area; BMC = bone mineral content; CHEU = children HIV-exposed uninfected; CHUU = children HIV-unexposed uninfected; HC = head circumference; HCAZ = HC-for-age SD-score; LAZ = length-for-age SD-score, LS = lumbar spine 1-4; MUAC = mid-upper arm circumference; MAZ = MUAC for-age SD-score; WB = whole body; WAZ = weight-for-age SD-score; WFLZ = weight-for-length SD-score; WK2, WK14, WK26, WK52, WK78 = 2, 14, 26, 52, 78 weeks of age, respectively.

Change within individuals by group **^a^***P* ≤0.001, **^b^***P* ≤0.01, **^c^**p≤0.05. Differences between groups from the same models are summarised in Table 4 and Supplement S6.

Supplement S8: Within individual changes between timepoints in Gumba infants by group: biochemical factors.

|  | WK2 to WK14 | | WK14 to WK26 | | WK26 to WK52 | | WK52 to WK78 | |
| --- | --- | --- | --- | --- | --- | --- | --- | --- |
|  | CHEU | CHUU | CHEU | CHUU | CHEU | CHUU | CHEU | CHUU |
| *Vitamin D status* |  |  |  |  |  |  |  |  |
| 25OHD, nmol/L | - | - | +6.2 (5.2) | +18.7 (5.1)**^a^** | -7.5 (5.4) | +4.7 (5.5) | -2.1 (5.9) | -0.04 (5.7) |
| *Hormones* |  |  |  |  |  |  |  |  |
| IGF1, % | - | - | -15.6 (12.5) | -13.7 (12.3) | -4.8 (12.9) | -6.7 (13.2) | +6.5 (14.0) | +15.4 (13.7) |
| IGFBP3, % | - | - | -0.3 (6.3) | +4.2 (6.2) | +2.4 (6.5) | -4.6 (6.6) | -2.1 (7.1) | +5.5 (6.9) |
| IGF1/IGFBP3, % | - | - | -15.3 (8.5)**^b^** | -17.9 (8.4)**^a^** | -7.2 (8.7) | -2.1 (8.9) | +8.6 (9.5) | +9.9 (9.2) |
| *Bone turnover markers* | |  |  |  |  |  |  |  |
| P1NP, % | - | - | -34.4 (13.7)**^a^** | -40.2 (13.5)**^a^** | -48.6 (14.1)**^a^** | -50.8 (14.5)**^a^** | -2.6 (15.4) | -16.7 (15.0) |
| CTX, % | - | - | +78.0 (24.6)**^a^** | +130 (24.2)**^a^** | +23.3 (25.3) | +19.1 (25.9) | -55.1 (27.6)**^b^** | -97.5 (26.8)**^a^** |
| P1NP/CTX, % | - | - | -112 (24.6)**^a^** | -170 (24.3)**^a^** | -72.0 (25.4)**^a^** | -70.0 (25.9)**^a^** | +52.5 (27.6)**^b^** | +80.8 (26.9)**^a^** |
| OC, % | - | - | +39.0 (17.4)**^a^** | +29.7 (17.2)**^b^** | -11.8 (17.9) | -22.0 (18.3) | -1.1 (19.5) | -2.8 (19.0) |

Data are mean within-individual changes (95% confidence intervals) from pairwise Scheffé post-hoc tests obtained in hierarchical models with timepoint, group, infant ID nested in group and a timepoint*group interaction term. 25OHD was modelled without logarithmic transformation and changes are in nmol/L. Data for hormones and bone turnover markers were transformed to natural logarithms before modelling and changes are presented as percentages. For models where the interaction term was not significant, removing the interaction term made no material effect to the *P*-values for group or timepoint. Data are for infants who were measured on ≥2 occasions, the values and numbers are given in Table 5.

Abbreviations: 25OHD = 25-hydroxyvitamin D; CHEU = children HIV-exposed uninfected; CHUU = children HIV-unexposed uninfected; CTX = C-terminal telopeptide; IGF1 = insulin-like growth factor-1; IGFBP3 = insulin-like growth factor binding protein-3; IGF1/IGFBP3 = IGF1/IGFBP3 molar ratio; OC = osteocalcin; P1NP = procollagen type 1 N-terminal-propeptide; WK2, WK14, WK26, WK52, WK78 = 2, 14, 26, 52, 78 weeks of age, respectively.

Change within individuals by group **^a^***P* ≤0.001, **^b^***P* ≤0.01, **^c^***P* ≤0.05. Differences between groups from the same models are summarised in Table 6.

Supplement S9: Differences between CHEU and CHUU with values at each timepoint: anthropometry and DXA measures

|  | WK2 | WK14 | WK26 | WK52 | WK78 | *P*interaction | *P*group | *P*timepoint |
| --- | --- | --- | --- | --- | --- | --- | --- | --- |
| *Anthropometry, CHEU=48, CHUU=53* | | |  |  |  |  |  |  |
| Weight, kg | -0.07 (0.22) | -0.42 (0.22)**^b^** | -0.38 (0.22)**^c^** | -0.48 (0.22)**^b^** | -0.62 (0.22)**^a^** | 0.02 | 0.01 | <0.0001 |
| Length, cm | -0.61 (0.75) | -1.72 (0.75)**^a^** | -1.44 (0.75)**^b^** | -1.75 (0.75)**^a^** | -2.26 (0.75)**^a^** | 0.04 | 0.0005 | <0.0001 |
| MUAC, cm | - | - | -0.26 (0.26) | -0.17 (0.26) | -0.05 (0.26) | 0.54 | 0.41 | <0.0001 |
| HC, cm | -0.02 (0.39) | -0.30 (0.39) | -0.26 (0.39) | -0.58 (0.39) | -0.74 (0.39)**^b^** | 0.10 | 0.12 | <0.0001 |
| *WB Bone measures, CHEU=54, CHUU=59* | | |  |  |  |  |  |  |
| BMC, g | +1.6 (4.0) | -3.6 (4.0) | -3.4 (4.0) | - | - | 0.13 | 0.59 | <0.0001 |
| BA, cm^2^ | +6.7 (11.2) | -14.6 (11.2)**^c^** | -19.1 (11.2)**^b^** | - | - | 0.004 | 0.33 | <0.0001 |
| aBMD, g/cm^2^ | +0.002 (0.004) | -0.001 (0.004) | +0.002 (0.004) | - | - | 0.72 | 0.67 | <0.0001 |
| *LS Bone measures, CHEU=60, CHUU=63* | | |  |  |  |  |  |  |
| BMC, g | -0.16 (0.09)**^b^** | -0.01 (0.09) | -0.07 (0.09) | - | - | 0.07 | 0.19 | <0.0001 |
| BA, cm^2^ | -0.42 (0.25)**^b^** | -0.41 (0.25)**^b^** | -0.26 (0.25) | - | - | 0.62 | 0.02 | <0.0001 |
| aBMD, g/cm^2^ | -0.007 (0.006)**^c^** | +0.006 (0.006) | -0.001 (0.006) | - | - | 0.007 | 0.81 | <0.0001 |
| *Body composition, CHEU=54, CHUU=59* | | |  |  |  |  |  |  |
| Lean mass, g | -21 (228) | -171(225) | -54 (225) | - | - | 0.37 | 0.70 | <0.0001 |
| Fat mass, g | +87 (263) | -126 (260) | -385 (260)**^c^** | - | - | 0.04 | 0.29 | <0.0001 |
| % Fat | +1.2 (3.3) | -0.1 (3.3) | -3.2 (3.3) | - | - | 0.16 | 0.67 | <0.0001 |

Footnote to Supplement S9

Data are mean differences (95% confidence intervals) from pairwise Scheffé post-hoc tests obtained in hierarchical models with timepoint, group, infant ID nested in group and a timepoint*group interaction term. For models where the interaction term was not significant, removing the interaction term made no material effect to the *P*-values for group or timepoint. Data are for infants who were measured on all occasions in each category.

Abbreviations: aBMD = areal bone mineral density; BA =bone area; BMC = bone mineral content; CHEU = children HIV-exposed uninfected; CHUU = children HIV-unexposed uninfected; HC = head circumference; LS = lumbar spine L1-4; MUAC = mid-upper arm circumference; WB = whole-body with head; WK2, WK14, WK26, WK52, WK78 = 2, 14, 26, 52, 78 weeks of age, respectively.

**^a,b,c^** Significance of difference between groups at each timepoint **^a^***P* ≤0.001, **^b^** *P* ≤0.01, **^c^***P* ≤0.05.

Supplement S10: Differences between CHEU and CHUU with values at each timepoint: anthropometric SD-scores

|  | WK2 | WK14 | WK26 | WK52 | WK78 | *P*interaction | *P*group | *P*timepoint |
| --- | --- | --- | --- | --- | --- | --- | --- | --- |
|  |  |  |  |  |  |  |  |  |
| WAZ | -0.19 (0.24) | -0.57 (0.24)**^a^** | -0.42 (0.24)**^c^** | -0.39 (0.24)**^c^** | -0.47 (0.24)**^b^** | 0.26 | 0.02 | <0.0001 |
| LAZ | -0.35 (0.32) | -0.87 (0.32)**^a^** | -0.60 (0.32)**^b^** | -0.58 (0.32)**^b^** | -0.71 (0.32)**^a^** | 0.20 | 0.001 | <0.0001 |
| WFLZ | +0.14 (0.38) | +0.12 (0.38) | -0.07 (0.38) | -0.12 (0.38) | -0.16 (0.38) | 0.73 | 0.92 | <0.0001 |
| MAZ | - | - | -0.21 (0.22) | -0.14 (0.22) | -0.09 (0.23) | 0.77 | 0.37 | 0.16 |
| HCAZ | -0.03 (0.36) | -0.29 (0.35) | +0.01 (0.35) | -0.35 (0.36) | -0.50 (0.37) | 0.25 | 0.22 | <0.0001 |

Data are mean differences (95% confidence intervals) from pairwise Scheffé post-hoc tests obtained in hierarchical models with timepoint, group, infant ID nested in group and a timepoint*group interaction term. For models where the interaction term was not significant, removing the interaction term made no material effect to the *P*-values for group or timepoint. Data are for infants who were measured on all occasions, CHEU = 48, CHUU = 53.

Abbreviations: CHEU = children HIV-exposed uninfected; CHUU = children HIV-unexposed uninfected; HCAZ = head circumference-for-age SD-score; LAZ = length-for-age SD-score; MAZ = mid-upper arm circumference-for-age SD-score; WAZ = weight-for-age SD-score; WFLZ = weight-for-length SD-score; WK2, WK14, WK26, WK52, WK78 = 2, 14, 26, 52, 78 weeks of age, respectively.

**^a,b,c^** Significance of difference between groups at each timepoint **^a^**p≤0.001, **^b^**p≤0.01, **^c^**p≤0.05.

Supplement S11: Differences between CHEU and CHUU with values at each timepoint: biochemical factors

|  | WK2 | WK14 | WK26 | WK52 | WK78 | *P*interaction | *P*group | *P*timepoint |
| --- | --- | --- | --- | --- | --- | --- | --- | --- |
| *Vitamin D status* |  |  |  |  |  |  |  |  |
| 25OHD, nmol/L | - | +31.0 (6.2)^a^ | +17.3 (6.2)^a^ | +4.4 (6.2) | +0.8 (6.2) | <0.0001 | 0.0002 | <0.0001 |
| *Hormones* |  |  |  |  |  |  |  |  |
| IGF1, % | - | -11.2 (15.4) | -9.0 (15.4) | -9.2 (15.4) | -13.4 (15.4) | 0.98 | 0.10 | 0.002 |
| IGFBP3, % | - | -6.7 (7.8) | -10.7 (7.8) | -3.4 (7.8) | -9.2 (7.8) | 0.59 | 0.06 | 0.76 |
| IGF1/IGFBP3, % | - | -4.4 (10.2) | +1.7 (10.2) | -5.8 (10.2) | -4.2 (10.2) | 0.74 | 0.50 | <0.0001 |
| *Bone turnover markers* | |  |  |  |  |  |  |  |
| P1NP, % | - | -3.8 (18.2) | -0.1 (18.2) | +0.1 (18.2) | +14.0 (18.2) | 0.55 | 0.67 | <0.0001 |
| CTX, % | - | +47.0 (31.5)^c^ | +10.5 (31.5) | -3.9 (31.5) | +37.7 (31.5) | 0.09 | 0.01 | <0.0001 |
| P1NP/CTX, % | - | -50.9 (31.5)^c^ | -10.7 (31.5) | +3.9 (31.5) | -23.8 (31.5) | 0.10 | 0.04 | <0.0001 |
| OC, % | - | -16.2 (22.8) | -7.2 (22.8) | +3.6 (22.8) | +4.5 (22.8) | 0.55 | 0.61 | <0.0001 |

Data are mean percentage differences (95% confidence intervals) from pairwise Scheffé post-hoc tests obtained in hierarchical models with timepoint, group, infant ID nested in group and a timepoint*group interaction term with data transformed to natural logarithms. 25OHD was modelled without logarithmic transformation and differences are in nmol/L. For models where the interaction term was not significant, removing the interaction term made no material effect to the *P*-values for group or timepoint. Data are for infants who were measured on all 4 occasions, CHEU=43, CHUU=51.

Abbreviations: 25OHD = 25-hydroxyvitamin D; CHEU = children HIV-exposed uninfected; CHUU = children HIV-unexposed uninfected; CTX = C-terminal telopeptide; IGF1 = insulin-like growth factor- 1; IGFBP3 = insulin-like growth factor binding protein 3; IGF1/IGFBP3 = IGF1/IGFBP3 molar ratio; OC = osteocalcin; P1NP = procollagen type 1 N-terminal-propeptide; WK2, WK14, WK26, WK52, WK78 = 2, 14, 26, 52, 78 weeks of age, respectively.

**^a,b,c^** Difference between groups at each timepoint **^a^***P* ≤0.001, **^b^***P* ≤0.01, **^c^***P* ≤0.05.
